# Topochemical distribution of lignin and hydroxycinnamic acids in sugar-cane cell walls and its correlation with the enzymatic hydrolysis of polysaccharides

**DOI:** 10.1186/1754-6834-4-7

**Published:** 2011-03-16

**Authors:** Germano Siqueira, Adriane MF Milagres, Walter Carvalho, Gerald Koch, André Ferraz

**Affiliations:** 1 Departamento de Biotecnologia, Escola de Engenharia de Lorena, Universidade de São Paulo, CP 116, 12602-810 Lorena, SP, Brasil; 2 Institute of Wood Technology and Wood Biology, Federal Research Institute for Rural Areas, Forestry and Fisheries, D-21031 Hamburg, Germany

## Abstract

**Background:**

Lignin and hemicelluloses are the major components limiting enzyme infiltration into cell walls. Determination of the topochemical distribution of lignin and aromatics in sugar cane might provide important data on the recalcitrance of specific cells. We used cellular ultraviolet (UV) microspectrophotometry (UMSP) to topochemically detect lignin and hydroxycinnamic acids in individual fiber, vessel and parenchyma cell walls of untreated and chlorite-treated sugar cane. Internodes, presenting typical vascular bundles and sucrose-storing parenchyma cells, were divided into rind and pith fractions.

**Results:**

Vascular bundles were more abundant in the rind, whereas parenchyma cells predominated in the pith region. UV measurements of untreated fiber cell walls gave absorbance spectra typical of grass lignin, with a band at 278 nm and a pronounced shoulder at 315 nm, assigned to the presence of hydroxycinnamic acids linked to lignin and/or to arabino-methylglucurono-xylans. The cell walls of vessels had the highest level of lignification, followed by those of fibers and parenchyma. Pith parenchyma cell walls were characterized by very low absorbance values at 278 nm; however, a distinct peak at 315 nm indicated that pith parenchyma cells are not extensively lignified, but contain significant amounts of hydroxycinnamic acids. Cellular UV image profiles scanned with an absorbance intensity maximum of 278 nm identified the pattern of lignin distribution in the individual cell walls, with the highest concentration occurring in the middle lamella and cell corners. Chlorite treatment caused a rapid removal of hydroxycinnamic acids from parenchyma cell walls, whereas the thicker fiber cell walls were delignified only after a long treatment duration (4 hours). Untreated pith samples were promptly hydrolyzed by cellulases, reaching 63% of cellulose conversion after 72 hours of hydrolysis, whereas untreated rind samples achieved only 20% hydrolyzation.

**Conclusion:**

The low recalcitrance of pith cells correlated with the low UV-absorbance values seen in parenchyma cells. Chlorite treatment of pith cells did not enhance cellulose conversion. By contrast, application of the same treatment to rind cells led to significant removal of hydroxycinnamic acids and lignin, resulting in marked enhancement of cellulose conversion by cellulases.

## Background

The recalcitrance of lignocellulosic materials to enzymatic hydrolysis is clearly associated with the limited porosity of lignified cell walls. This limited porosity hinders enzyme infiltration into non-pretreated lignocellulosic materials [1-5]. Lignin and hemicelluloses are the major components limiting enzyme infiltration into the cell walls. These components also encapsulate cellulose microfibrils, preventing the access of cellulases to the cellulose chains [6,7]. In grasses, the hydroxycinnamic acid content is an important influence on recalcitrance [8]. In such monocotyledons, the various chemical compositions and structures of the individual cell types make elucidation of the recalcitrance in these plant materials more difficult [3,9].

Cellular ultraviolet (UV) microspectrophotometry (UMSP) has been used to provide information on the topochemical distribution of lignin and other aromatic compounds in diverse cell walls [10-14]. Recently, Akin [9] correlated the topochemistry of lignin and aromatics with the biodegradability of cell walls in various lignocellulosic materials.

There have as yet been no reports of attempts to correlate the topochemical distribution of lignin and hydroxycinnamic acids in sugarcane cell walls with the *in vitro *recalcitrance of such material to hydrolytic enzymes. Taking into account the cell-wall characteristics of sugar cane, it is clear that lignin represents one of the main hindrances to cellulosic ethanol production from this lignocellulosic material. Determination of the topochemical distribution of lignin and aromatics in sugar cane might also provide important data on the recalcitrance of specific cells, which would be useful for research programs aiming to improve cultivars and design mild pretreatment processes for efficient biomass conversion.

Sugar cane is a monocotyledon with a complex cellular anatomy, comprised mainly of vascular bundles and parenchyma cells. In a sugar-cane internode, the outermost part is named the rind and the inner region is usually named the pith. The rind contains minor amounts of parenchyma cells and most of the vascular bundles, which are formed by vessels surrounded by a large number of fibers. The pith is rich in sucrose-storing parenchyma cells, but also contains small numbers of vascular bundles [15]. Vessel and parenchyma cells have a wide lumen and relatively thin cell walls, whereas fibers are thick-walled and have a narrower lumen [16].

The distribution of lignin and hydroxycinnamic acids in the different morphological regions of sugar cane was previously studied by He and Terashima [17,18], using microautoradiography and UMSP. These authors found that syringyl-propane, guaiacyl-propane and *p*-hydroxyphenyl-propane lignin subunits various between fiber, vessel and parenchyma cell walls. Hydroxycinnamic acid residues could also be detected by UMSP, because their wavelengths of maximum absorption (315-320 nm) differed from those of lignin (278-282 nm). Lignification of vessels was found to occur in the early cell maturation stage followed by lignification of fibers. Sinapyl acid, *p*-coumaric acid and ferulic acid were shown to deposit at the early stage of lignification, and the levels of hydroxycinnamic acids were higher in parenchyma walls than in the vascular bundles.

In this study, we attempted to elucidate the factors related to lignin hindrance for enzymatic hydrolysis of sugar-cane lignified cells. For topochemical analyses, cellular UMSP was used to provide a complete view of the distribution of lignin and hydroxycinnamic acids in the individual cell wall types. Model sugar-cane substrates depleted in lignin were also prepared by aqueous acetic acid/chlorite delignification [19]. These samples were then treated with commercial cellulases to evaluate the effects of lignin and hydroxycinnamic acids on the enzymatic hydrolysis of polysaccharides.

## Results and discussion

### Sugar-cane cell anatomy and topochemical distribution of aromatic components

**Figure 1:**
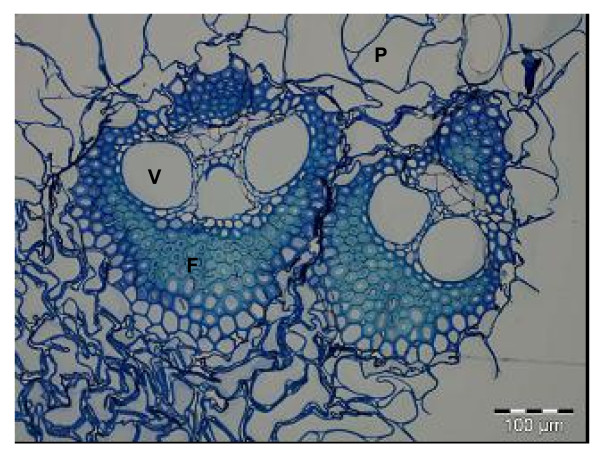
**Transverse section from the rind region of a sugar-cane internode after toluidine blue staining**. The typical cell types are indicated: V = vessel, F = fibers and P = parenchyma.

Rind and pith regions were excised from sugar cane internodes, cut into transverse sections 1 μm thick, and examined by microscopy. Using toluidine blue staining, we found that the sugar-cane internodes had the typical vascular bundles (vessels and surrounding fibers) and sucrose-storing parenchyma cells (Figure 1). The vascular bundles were more abundant in the rind, whereas parenchyma cells predominated in the pith region, consistent with the literature on sugar-cane anatomy [15] and other grasses as revised recently [9]. These cell types are well described in the literature, and are characterized by various lengths, diameters and wall thicknesses [15,16].

**Figure 2:**
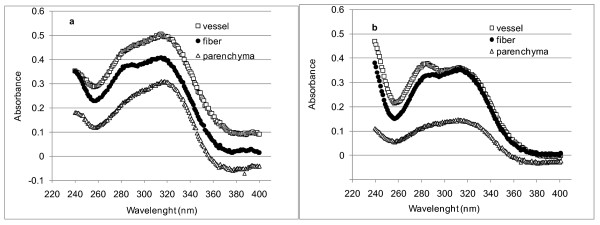
**Representative UV spectra of individual cell walls (S2) of untreated sugar-cane, showing **(A) **rind and **(B) **pith S2, respectively**. At least five spectra were recorded from each different cell type. The average spectra are shown in this figure. Standard deviations, calculated from the absorbance values measured at the wavelength of maximal absorption, were in the range of 5%, 13% and 25% for fiber, vessel and parenchyma cells, respectively.

UMSP was used to record UV spectra from spots 1 μm^2 ^in size located in the secondary wall (S2) of the three main cell types (Figure 2). The highest UV absorbance was measured in the S2 of vessels from rind and pith, followed by fibers and parenchyma. UV spectra of fiber and vessel S2 walls had defined bands near 278 nm and 315 nm (Figure 2). The band at 278 nm is produced by the aromatic rings in guaiacyl lignin, whereas the strong band at 315 nm is typical of hydroxycinnamic acids linked to the lignin and/or the arabino-methylglucurono-xylan backbones often found in grasses [9,18,20]. The spectra from the S2 of parenchyma cell walls had the lowest absorbance values, especially in the pith region. The band at 278 nm was not resolved in these spectra, but a pronounced band appeared at 315 nm, which is consistent with the predominance of hydroxycinnamic acids esterified to arabino-methylglucurono-xylans [9,21].

**Figure 3:**
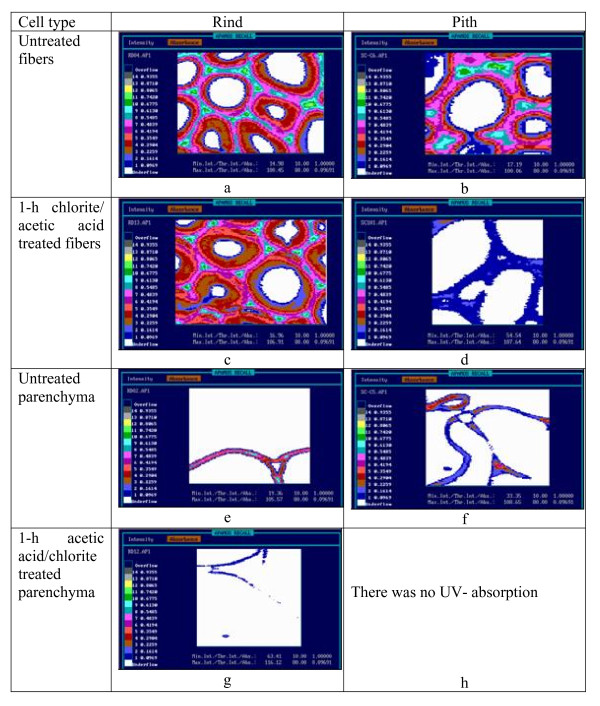
**Scanning ultraviolet (UV) micrographs of 1-μm transverse sections of sugar-cane cells**. The colored pixels represent different UV absorbance values of the cell-wall layers measured at 278 nm with a geometric spatial resolution of 0.25 × 0.25 μm per pixel.

Selected areas of the sugar-cane tissues were further scanned at constant wavelengths of 278 and 315 nm with a spatial resolution of 0.25 μm. The signals of the UV-spectrophotometer were converted into digitized images (APAMOS software; Zeiss, Jena, Germany). Colored micrographs for selected samples are illustrated in Figure 3 (see Methods). The scanned micrographs of fiber tissues from the rind and the pith had similar absorbance levels, with the most intense absorbance seen in the cell corners (CC) and the compound middle lamella (CML). Frequency histograms from each image were evaluated to calculate average absorbance values [22]. In the rind and pith fibers, the average absorbance values at 278 nm were 0.40 and 0.39 (including all cell-wall layers), respectively, whereas, those of the parenchyma tissue were significantly lower at 0.31 and 0.18, respectively. Comparing the results obtained from the spectral point measurements and scanning analyses for the individual cell types and layers, both datasets had very good congruence (compare Figure 2 and Figure 3).

Excised rind from untreated sugar cane contained 19% lignin, 30% hemicelluloses and 44% cellulose, whereas the pith fraction had corresponding values of 12%, 24% and 53%. Considering that parenchyma cells predominate in the pith region, these data are in close agreement with those of He and Terashima [17,18], who reported lower lignin content in the parenchyma compared with the vascular bundle tissues. Notably, part of the lignin measured by the Klason procedure actually corresponds to the hydroxycinnamic acids that condense with lignin during the analytical acid treatment [23].

Treatment of the rind and pith samples with aqueous acetic acid/chlorite caused selective removal of lignin and hydroxycinnamic acids. Total lignin contents decreased rapidly, from 19% to 7% after 4 hours of treatment for rind samples, and from 12% to 7% after 2 hours of treatment for pith samples. Selected portions of the treated sugar-cane tissues were also examined by the UV spectroscopic techniques. The lignin (and accessory aromatic compounds) originally deposited in the fiber cell walls of the rind tissue mainly resisted a delignification process of 1 hour, whereas the pith fiber tissue was rapidly delignified (Figure 3a-d). In fact, the rind fibers required prolonged treatment (4 hours) for significant removal of the aromatic components. By contrast, the cell walls of the rind parenchyma tissue displayed a distinct decrease in UV absorbance values after 1 hour of treatment with aqueous acetic acid/chlorite (Figure 3e,g). The highest level of delignification was found in the cell walls of the pith parenchyma tissue (Figures 3f,h); after a treatment of 1 hour, none absorbance values were quantified using the highly-sensitive UMSP technique. The studied vessel cell walls were characterized by heterogeneous delignification (measured at 278 nm, representing guaiacyl lignin), but the absorbance at 315 nm decreased significantly for both rind and pith cells (data not shown).

**Figure 4:**
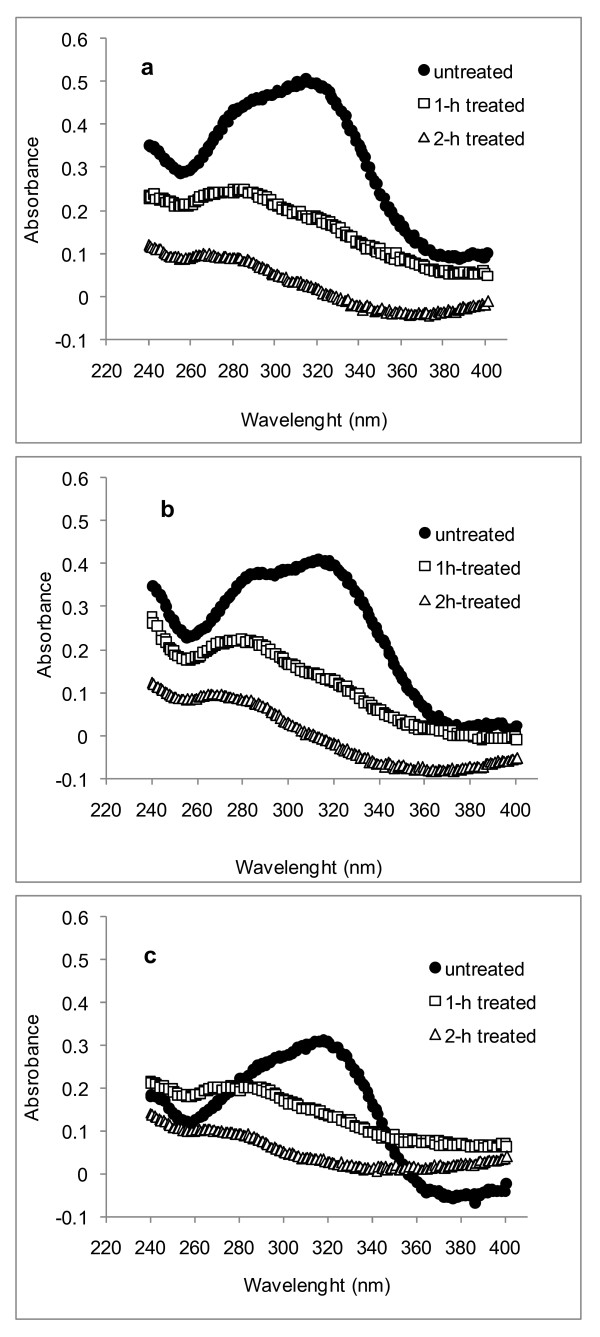
**Representative ultraviolet (UV) absorbance spectra of individual rind cell walls (S2) of sugar cane delignified under aqueous acetic acid/chlorite solution**. **(A) **Vessel, **(B) **fiber and **(C) **parenchyma cells, respectively. At least five spectra were recorded for each different cell type, and the average of each is shown.

**Figure 5:**
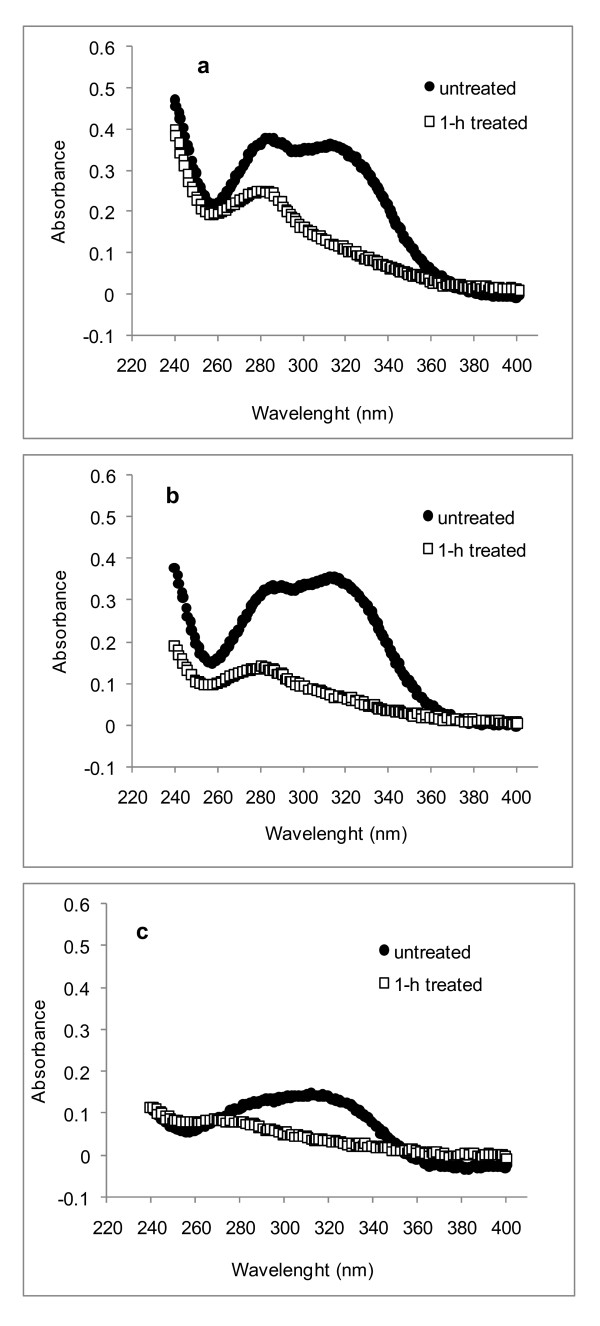
**Representative ultraviolet (UV) absorbance spectra of individual pith cell walls (S2) of sugar cane delignified under aqueous acetic acid/chlorite solution**. **(A) **Vessel, **(B) **fiber and **(C) **parenchyma cells, respectively. At least five spectra were recorded from each different cell type, and the average of each is shown.

UV spectra obtained from the S2 of fibers, vessels and parenchyma cells of the aqueous acetic acid/chlorite-treated samples are shown in Figure 4 and Figure 5. Treatment time was extended up to 4 hours, but in general, the removal of lignin and hydroxycinnamic acids was fully completed after 2 hours. After this time, the remaining lignin (detected by the topochemical analysis) was located in the compound middle lamella and cell corners. Evaluation of the S2 UV spectra of all cell types revealed that the hydroxycinnamic acids were the first to be removed, because the 315 nm band decreased in intensity more rapidly than did the bands at 278 nm (Figure 4, Figure 5). In pith cells, removal of hydroxycinnamic acids was even more pronounced (Figure 5).

### Enzymatic hydrolysis of rind and pith samples

**Figure 6:**
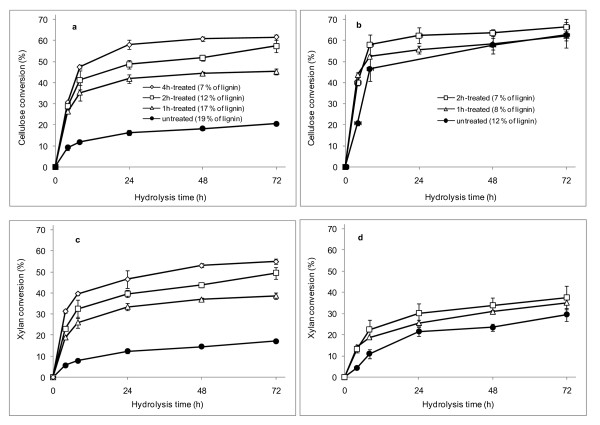
**Cellulose and xylan conversion to monosaccharides after enzymatic hydrolysis of **(A,C) **rind and **(B,D) **pith samples treated with aqueous acetic acid/chlorite**.

The untreated rind and pith samples and the aqueous acetic acid/chlorite-treated samples were digested with a mixture of cellulolytic enzymes. Beside cellulose, xylan present in the bagasse (fibre) samples was also hydrolyzed by the enzymatic cocktail (Figure 6a-d). The xylan hydrolysis was a result of the xylanase and β-xylosidase activities present in the commercial enzyme preparations [24-26]. For rind samples, the xylan conversion to xylose was similar to that seen for cellulose hydrolysis, but for pith samples, it was significantly lower than the cellulose hydrolysis (Figure 6b,d), indicating that the hemicellulose remaining in the pith fraction after the delignification was more recalcitrant to enzymatic attack.

Cellulose from untreated pith samples was promptly hydrolyzed to glucose, reaching 63% conversion after 72 hours of hydrolysis, whereas untreated rind samples had only 20% conversion, indicating that the less lignified parenchyma cells, which mainly occurred in the pith region, were significantly less recalcitrant than the fibers and vessels predominating in the rind region. This is consistent with previous work showing that parenchyma cells from maize stems at various states of maturation were preferentially degraded by rumen biota [27]. Hansen *et al*. [28] also reported that parenchyma cells surrounding the pith cavity lining of thermally pretreated wheat straw were promptly hydrolyzed by a mixture of cellulolytic enzymes. Akin revised the histochemical findings for degradation of various tissues by cellulolytic systems, and confirmed that cells with lower levels of lignin are the first to be hydrolyzed in several plant species [9].

The UMSP data showed that parenchyma cells in the pith region are characterized by the lowest UV-absorbance values, mostly related to hydroxycinnamic acids, because the measured S2 spectrum had its peak only at 315 nm. Taken together, these results suggest that the action of the cellulolytic enzymes was not restrained by the aromatics occurring in the pith parenchyma. In addition, aqueous acetic acid/chlorite treatment of pith did not enhance the cellulose or xylan conversion, corroborating the notion that the recalcitrance of this fraction does not depend on the presence of aromatics only. It is likely that the limited hydrolysis of xylan to xylose also reflects the hindrance to cellulase action, as the hemicelluloses are known to encapsulate cellulose fibrils in the cell walls [3]. More recently, Qing and Wyman [25] found that xylo-oligomers accumulating during enzymatic treatment of xylans can also inhibit cellulolytic enzymes. In our study, we found that partially delignified fiber and vessels remained in the pith fraction, and might also account for the maximum level of cellulose conversion of around 63-66% (Figure 6b). Conversely, treatment of the rind cells with aqueous acetic acid/chlorite, which led to significant removal of hydroxycinnamic acids and lignin (Figure 4), resulted in significant enhancement of cellulose and xylan conversion by the commercial cellulases (Figure 6a).

**Figure 7:**
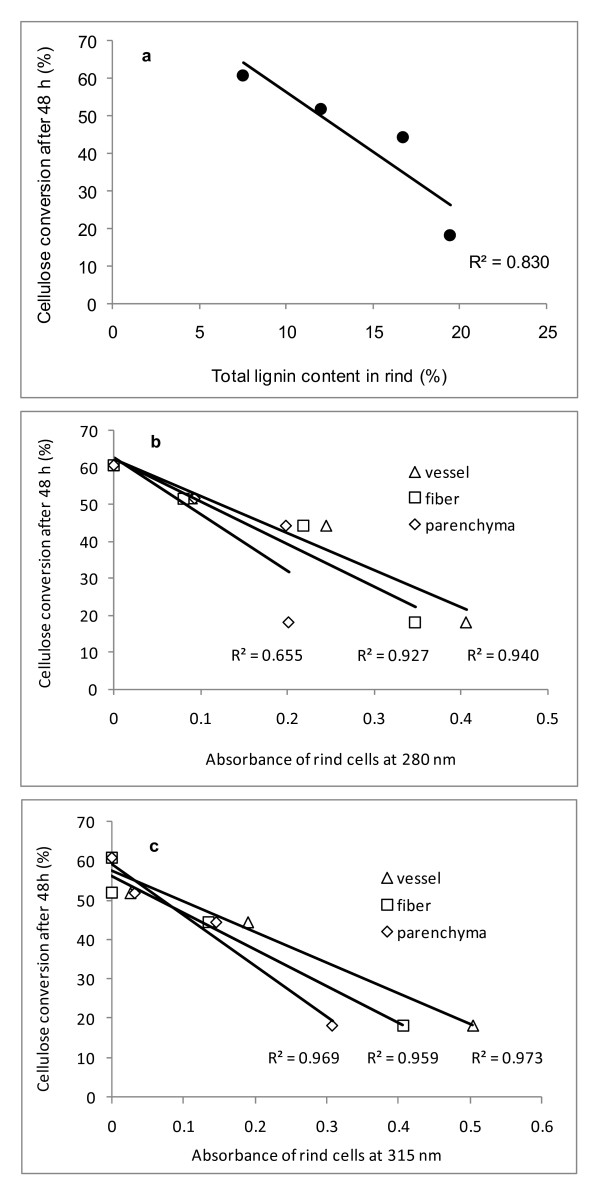
**Cellulose conversion by cellulolytic enzymes as a function of lignin content or **(B,C) **absorbance data of individual tissues at **(B) **280 nm and **(C) **315 nm for rind cells from sugar cane**.

There was an inverse correlation between cellulose conversion levels and lignin contents or absorbance data for the various tissue types in rind (Figure 7). Absorbance data at 280 nm and 315 nm (Figure 4, Figure 5) were used as an estimate of lignin and hydroxycinnamic acid contents, respectively, in each cell type. The *r*^2 ^values for data correlation by linear models were relatively high (Figures 7b,c). An exception was the data for rind parenchyma at 280 nm, which gave an *r*^2 ^value of only 0.655. For the total lignin detected in the rind fraction, the correlation with cellulose conversion was also high (*r*^2 ^= 0.83), reflecting the weighted average of absorbances at 280 nm of each individual cell type. The inverse correlation seen for the lignin content or the absorbance assigned to aromatics with the levels of cellulose conversion is in close agreement with data reported in previous studies by Grabber *et al*. [29] for mature and immature alfalfa fibers, Jung and Casler [27] for maize stems at various stages of maturation, Chen and Dixon [30] for transgenic alfalfa, and Lee *et al*. [31] for selectively delignified wood. With sugar-cane bagasse, we also found previously that a mild chemo-thermo-mechanical pretreatment led to enhanced cellulose conversion by cellulases, which was due to lignin removal during the pretreatment [26]. In this study, these correlations were not noticeable for pith samples, because the untreated fraction was promptly hydrolyzed by the cellulases to a cellulose conversion level of 63-66%, and treatment with acetic acid/chlorite did not enhance this conversion efficiency.

## Conclusions

Cellular UMSP was used to provide a complete view of the distribution of lignin and hydroxycinnamic acids in the individual cell-wall types of sugar-cane substrates. UV spectra from 1 μm^2 ^spots located in the S2 of the three main cell types showed that the highest UV absorbance occurred in the S2 of vessels from rind and pith, followed by fibers and parenchyma. The spectra from the S2 of parenchyma cell walls gave the lowest absorbance values, especially in the pith region. The band at 278 nm was not resolved in these spectra, whereas a pronounced band appeared at 315 nm, which is consistent with the predominance of hydroxycinnamic acids esterified to arabino-methylglucurono-xylans. Untreated pith samples were promptly hydrolyzed by cellulases, indicating that the less lignified parenchyma cells, which mainly occurred in the pith region, were significantly less recalcitrant than the fibers and vessels predominating in the rind region. Chlorite treatment of pith cells did not enhance cellulose conversion, whereas in rind cells it led to significant removal of hydroxycinnamic acids and lignin, resulting in a marked enhancement of the cellulose conversion by cellulases.

## Methods

### Sugar-cane sampling and preparation

One single stalk of a mature (1.5 years old) sugar-cane plant was cut by hand from a plantation located in Lorena, SP, Brazil. Because of the variation that can occur between sugar-cane varieties or even within the same population, our quantitative observations should be taken only as an approximation for other sugar-cane varieties. Internodes 3 to 7 (from the plant base) were separated and cut on the longitudinal axis into circular pieces 25 mm long. These pieces were cut as follows: 1) the first external 2 mm area containing the bark and epidermis was discarded; 2) one fragment 11 mm in diameter was excised from the center region, using a laboratory cork-driller, which corresponded to the pith sample; 3) the next ring (9 mm thick) from the center to the border was cut out and discarded to avoid the pith and rind mixing in the samples; and 4) the remaining ring, approximately 10 mm thick, was further cut into long chips measuring 10 × 10 × 25 mm, corresponding to the rind samples.

The rind and pith samples were extracted with water in Soxleth apparatus (Provermex, SP, Brazil) using cycles of 8 hours to remove sucrose. After each extraction cycle, total sugars were assayed in the extract using the phenol-sulfuric acid assay. Extraction cycles were performed with fresh water until the extract became free of soluble sugars (in general, this took five cycles).

Extracted material was delignified at 75-80°C in aqueous sodium chlorite/acetic acid reagent for up to 4 hours [19]. The solid:liquid ratio was 1:26 (w/v), and the acetic acid and sodium chlorite loads were 0.1 ml/g and 0.3 g/g, respectively (oven-dried). At intervals of 1 hour, fresh acetic acid and sodium chlorite was added to the reaction tube. To avoid collapse of the sugar-cane tissues after long delignification periods, fishing line was tied around the cylindrical outer surface of the rind and pith pieces. After delignification, the samples were washed with acetone and water. The washed samples were placed into screw-capped tubes and maintained at 4°C until they were prepared for UMSP analyses and enzyme digestion assays.

### UMSP of the samples

For the UV spectroscopic analysis, small blocks (approximately 1 × 1 × 0.5 mm) were dissected from the rind and pith samples. The specimens were dehydrated in a graded series of acetone and embedded in Spurr's epoxy resin [32]. Sections 1 μm thick were prepared from these samples with a diamond knife, transferred to quartz microscope slides, and embedded in glycerine [33]. The topochemical analysis was carried out using a microspectrophotometer (UMSP 80; Zeiss) equipped with a scanning stage enabling the determination of image profiles at defined wavelengths using the scan program APAMOS (Zeiss). Wavelengths of 278 and 315 nm were selected for detection of the distribution of lignin or hydroxycinnamic acids, respectively. The scan program digitizes rectangular fields of the tissue with a local geometrical resolution of 0.25 × 0.25 μm and a photometrical resolution of 4,096 grayscale levels, which are converted into 16 basic colors to visualize the absorbance intensities. The samples were also investigated conventionally by point measurements with a spot size of 1 μm^2^, between 240 and 400 nm, using UVMSP (Tidas MSP 80, J&M), (J&M, Essingen/Aalen, Germany). At least five spectra were recorded from each individual cell type and cell-wall layer. The average absorbance values of the spectra are presented throughout the paper. Standard deviations, calculated from the absorbance measured at the wavelength of maximal absorption, were in the range of 5%, 13% and 25% for fiber, vessel and parenchyma cells, respectively.

### Determination of the chemical composition of the samples

The chemical composition of the lignocellulosic samples was determined in the ethanol-extracted material. Approximately 3 g of milled sample was extracted with 95% ethanol for 6 hours in a Soxleth extractor. Extracted samples (300 mg) were hydrolyzed with 72% (w/w) sulfuric acid (3 mL) at 30°C for 1 hour. The acid was diluted to a final concentration of 3% with the addition of 79 mL of water, and the mixture was heated at 121°C and 1 atm for 1 hour. The resulting material was cooled and filtered through a porous glass filter (number 3). The solids were dried to a constant weight at 105°C, and considered the insoluble lignin. The soluble lignin concentration in the filtrate was determined by measuring the absorbance at 205 nm and using the value 105 l/g/cm as the absorptivity of soluble lignin. Concentrations of monomeric sugars in the soluble fraction were determined by high-performance liquid chromatography (HPLC) (HPX87H column; Bio-Rad Laboratories, Richmond, CA, USA) at 45°C, eluted at a rate of 0.6 ml/min with 5 mmol/l sulfuric acid. Sugars were detected using a temperature controlled refractive index detector [34]. Because of the limited amounts of each sample, an estimate of experimental deviations in chemical compositions was based on triplicate repeats performed with the untreated rind samples. The glucan, hemicelluloses and total lignin contents varied by 2.1%, 2.3% and 1.0% of the average values, respectively.

### Enzymatic hydrolysis of the samples

The pith and rind samples were milled to pass through a 0.84 mm screen and digested with a mixture of commercial enzyme preparations (Celluclast and Novozym 188; Novozymes Biopharma DK, Bagsvaerd, Denmark) at a dosage of 10 filter-paper units (FPU) of cellulase and 20 IU of β-glucosidase per gram of substrate (oven-dried weight). Each hydrolysis experiment was carried out in 50-mL centrifuge tubes containing 200 mg of lignocellulosic material (oven-dried weight) and 10 mL of 50 mmol/L sodium acetate buffer pH 5.0, plus the enzyme solution (final consistency of 2%). The tubes were incubated at 45°C under reciprocal agitation of 120 cycles per min. At defined periods, from 4 to 72 hours, a sample of 250 μL was taken from each tube and heated to 100°C for 5 min, followed by spinning in a centrifuge at 11,260 g for 15 minutes. For each reaction time, three replicate experiments were carried out. Hydrolysates were assayed for glucose, cellobiose and xylose contents using the previously described HPLC procedure. Cellulose conversion was calculated as 0.9 × mass of glucose + 0.95 × mass of cellobiose released from the glucan contained in the sample. Only minor amounts of cellobiose were detected in the hydrolysates. Variation between hydrolysis triplicates is shown as standard deviation bars in the corresponding figures.

## Competing interests

The authors declare that they have no competing interests.

## Authors' contributions

GS performed the delignification experiments, enzymatic hydrolysis of samples, chemical analyses and data interpretation. AMFM participated in the design of the study, data interpretation and drafting of the manuscript. WC participated in the design of the study and data interpretation. GK participated in the cellular UMSP analyses, data interpretation and drafting of the manuscript. AF conceived the study, performed the delignification experiments, cellular UMSP analyses and data interpretation, and reviewed the manuscript. All authors read and approved the final manuscript.
